# Effect of Renal Function Impairment on the Mortality of Cirrhotic Patients With Hepatic Encephalopathy: A Population-Based 3-Year Follow-Up Study

**DOI:** 10.1097/MD.0000000000000079

**Published:** 2014-09-19

**Authors:** Tsung-Hsing Hung, Chih-Wei Tseng, Kuo-Chih Tseng, Yu-Hsi Hsieh, Chih-Chun Tsai, Chen-Chi Tsai

**Affiliations:** Division of Gastroenterology (T-HH, C-WT, K-CT, Y-HH), Department of Medicine, Dalin Tzu Chi Hospital, Buddhist Tzu Chi Medical Foundation, Chiayi; School of Medicine (T-HH, C-WT, K-CT, Y-HH, C-Chi Tsai), Tzu Chi University, Hualien; Department of Mathematics (C-Chun Tsai), Tamkang University, Tamsui; Division of Infectious Disease (C-Chi Tsai), Department of Medicine, Dalin Tzu Chi Hospital, Buddhist Tzu Chi Medical Foundation, Chiayi, Taiwan.

## Abstract

Kidney is an important organ to clear neurotoxic substance in circulation. However, it is still unknown about the effect of renal function impairment (RFI) on the mortality of cirrhotic patients with hepatic encephalopathy (HE). We used the Taiwan National Health Insurance Database to identify 4932 cirrhotic patients with HE, hospitalized between January 1, 2007 and December 31, 2007. The enrolled patients were followed up individually for 3 years to identify their 3-year mortalities. There were 411 (8.3%) patients with RFI and 4521 (91.7%) patients without RFI. The adjusted hazard ratio (HR) of RFI for 3-year mortality was 2.03 (95% CI, 1.82–2.27). In RFI group, there were 157 (38.2%) patients with acute renal failure (ARF), 61 (14.8%) with hepatorenal syndrome (HRS), 93 (22.6%) with chronic kidney disease (CKD), and 100 (24.3%) with end-stage renal disease (ESRD). Compared with the non-RFI group, the adjusted HR of ARF for 3-year mortality was 2.57 (95% CI, 2.17–3.06), CKD 1.93 (95% CI, 1.55–2.40), ESRD 1.26 (95% CI, 1.01–1.57), and HRS 3.58 (95% CI, 2.78–4.63). Among ESRD patients, there were 99 patients receiving hemodialysis regularly. Compared with the CKD group, the adjusted HR of ESRD with hemodialysis for 3-year mortality was 0.664 (95% CI, 0.466–0.945). RFI increased the 3-year mortality of cirrhotic patients with HE, especially ARF and HRS. HE patients with ESRD receiving hemodialysis had better 3-year survival rate than those with CKD.

## INTRODUCTION

In decompensated cirrhosis, the endogenous neurotoxic substances, such as ammonia, glutamine, methionine, serotonin, benzodiazepines, and gamma-amino-butyric acid, escape catabolism from the liver. Elevated neurotoxic substances in systemic circulation pass through blood–brain barriers and reach brain parenchyma to impair cerebral function, which is called hepatic encephalopathy (HE).^1,2^ The incidence of HE was 2%–20% per year in patients with decompensated cirrhosis.^3,4^ Once cirrhotic patients have HE, their prognosis rapidly worsens. Without liver transplantation, their 1-year survival rate is 40%–50%, and the 3-year survival rate is only 23%.^5,6^

The common precipitating factors of HE included infection, gastrointestinal bleeding, and constipation.^6–10^ Kidney is an important organ to clear bloodstream ammonia that increases the susceptibility to brain edema.^11–13^ In previous studies, acute renal failure (ARF) is an unfavorable prognostic factor for 30-day mortality of HE patients.^6,7^ However, other forms of renal function impairment (RFI), including end-stage renal disease (ESRD), chronic kidney disease (CKD), and hepatorenal syndrome (HRS), has not been well studied, and their effects on the mortality of HE patients have still been inconclusive. We used Taiwan’s nationwide population-based dataset to enroll a large population of cirrhotic patients with HE. All patients were followed up individually to determine their 3-year mortality and evaluate the effects of various forms of RFI on their mortalities.

## MATERIALS AND METHODS

### Ethics Statement

This study was initiated after the approval by the Institutional Review Board of Buddhist Dalin Tzu Chi Hospital, Chiayi, Taiwan. Since all identifying personal information was removed from the secondary files prior to analysis, the review board waived the requirement for written informed consent from the patients whose data were involved.

### Database

This de-identified database used in this study was from the National Health Insurance Research Database (NHIRD), Taiwan, established and maintained by the Taiwan National Health Insurance Bureau and the National Health Research Institute. The dataset in this study is from this database, which includes all diagnostic coding information of the hospitalized patients in Taiwan. The Taiwan National Health Insurance Program was developed in 1995 and included all citizens living in Taiwan; this program covers more than 99% of Taiwan’s population. Many articles have been published based on the NHIRD.^14,15^ The privacy of the patients was protected, and all the study protocols needed to be evaluated by the National Health Research Institute. This study was approved by the National Health Research Institute (application and agreement number 101516).

### Study Sample

This retrospective study included the patients discharged with the diagnosis of cirrhosis (International Classification of Diseases, 9th Revision, Clinical Modification code [ICD-9-CM] 571.5, or 571.2 in the database) between January 1, 2007 and December 31, 2007. In this situation, the cirrhotic patients with HE (ICD-9-CM code 572.2) were enrolled for evaluation.^16^ In the cases of multiple hospitalizations, only the first episode was included. The patients receiving liver transplantation were excluded. Finally, a total of 4932 cirrhotic patients with HE were enrolled. They were divided into RFI group and non-RFI group. They were followed up individually for 3 years to identify their 3-year mortalities. The patients with RFI were defined as those with diagnosis codes related to RFI (ICD-9-CM codes 584, 585, 586, and 572.4, or other procedure codes related to renal failure).^17^

We stratified the RFI patients into four groups, including ESRD, ARF, HRS, and CKD. ESRD was defined as the patients receiving long-term hemodialysis or peritoneal dialysis before admission for HE. In our country, the patients with long-term dialysis had a certification to reduce their medical payment. An isolated dataset from the NHIRD could be used to identify the patients with long-term dialysis. HRS was defined as a patient with the ICD-9-CM diagnosis code of 572.4. ARF was defined as a patient with the ICD-9-CM diagnosis codes of 584, and without 572.4. Except for the patients with ESRD, HRS, or ARF, the patients with the ICD-9-CM diagnosis code of 585 were identified as the patients with CKD, not requiring long-term dialysis before admission for HE. Because the ICD-9-CM diagnosis code of 586 is an obscure code for renal failure, we reviewed the other diagnosis and procedure codes for those patients to identify if they had ARF, CKD, HRS, or ESRD. In order to reduce the possibility of wrong coding or classification, we reviewed all diagnosis and procedure codes in every hospitalization before the time of enrolling in all enrolled RFI patients.

In order to regress the effect of RFI on the mortality of cirrhotic patients with HE, we selected the mortality-related factors as comorbid medical factors, including alcoholism (ICD-9-CM codes 291, 303, 305.00–305.03, 571.0–571.3), hepatocellular carcinoma (HCC) (ICD-9-CM code 155.0), peptic ulcer bleeding (PUB) (ICD-9-CM codes 531.0, 531.2, 531.4, 531.6, 532.0, 532.2, 532.4, 532.6, 533.0, 533.2, 533.4, and 533.6), esophageal variceal bleeding (EVB) (ICD-9-CM codes 456.0, 456.20), ascites (ICD-9-CM code 789.5, or procedure code 54.91), and bacterial infections. The bacterial infections included pneumonia (ICD-9-CM codes 481–487, without 484),^18^ liver abscess (ICD-9-CM code 572.0), empyema (ICD-9-CM code 510), cellulitis (ICD-9-CM code 681 or 682), necrotizing fasciitis (ICD-9-CM code 728.86), central nerve system infection (including bacterial meningitis or brain abscess; ICD-9-CM code 324 or 320), sepsis (ICD-9-CM codes 038, 020.0, 790.7, or 112.81),^19^ infective endocarditis (ICD-9-CM code 421), urinary tract infection (ICD-9-CM code 590.1, 595.0, 595.9, or 599.0),^20^ biliary tract infection or acute cholecystitis (ICD-9-CM codes 576.1, 575.0, 574.00, 574.01, 574.30, 574.31, 574.60, 574.61, 574.80, and 574.81), septic arthritis (ICD-9-CM code 711), perianal abscess (ICD-9-CM code 566), and spontaneous bacterial peritonitis. Spontaneous bacterial peritonitis was defined as a patient with the ICD-9-CM diagnosis code 567.2, 567.8, or 567.9, and without the procedure codes for the abdominal surgery.^21,22^ In addition to the diagnosis codes mentioned above, we checked all diagnosis codes of the enrolled patients to confirm if the patients have bacterial infections.

### Statistical Analyses

The SPSS statistical package (SPSS System for Windows, version 12.0, SPSS Inc, Chicago, IL) was used for statistical analysis. The Student *t* test was used to compare continuous variables, and the chi square test was used to compare categorical variables. In order to identify risk factors for the mortality, the proportional hazards Cox regression model was used to control for possible confounding factors. We present hazard ratios (HRs) with the 95% confidence intervals (CIs) using a significance level of *P* value <0.05. The starting point to evaluate the 3-year mortalities in the cirrhotic patients with HE was the admission date of the enrolled hospitalization.

## RESULTS

Of the total 4932 cirrhotic patients with HE, the mean age was 57.2 ± 14.1 years and 3499 (71.0%) were male. The demographic characteristics of HE patients with and without RFI are listed in Table 1. There were 411 patients with RFI and 4521 patients without RFI. The patients with RFI were older than those without RFI. More patients were male and alcohol related in the non-RFI group. The overall 30-day, 90-day, 1-year, and 3-year mortalities in the RFI group were 36.6%, 59.5%, 76.6%, and 89%, respectively. The overall 30-day, 90-day, 1-year, and 3-year mortalities in the non-RFI group were 16.4%, 28.3%, 49.1%, and 70.1%, respectively (*P* < 0.001).

**TABLE 1:**
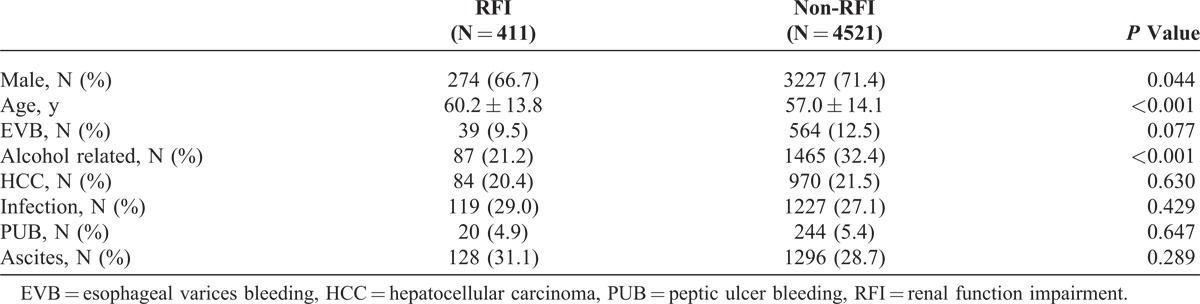
Demographic Characteristics of Cirrhotic Patients Having Hepatic Encephalopathy With and Without Renal Function Impairment

**FIGURE 1:**
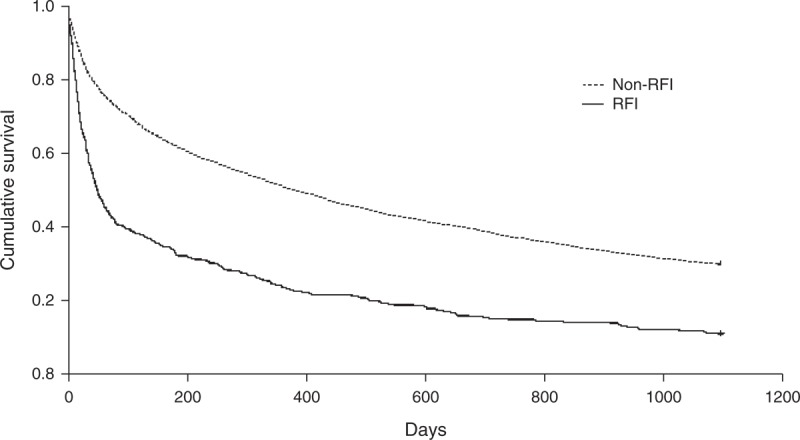
Cumulative survival plot for cirrhotic patients having hepatic encephalopathy with and without renal function impairment.

The results of Cox regression analysis of HR for the predisposing factors of 3-year mortality are provided in Table 2. After adjusting for the patients’ gender, age, and other comorbid disorders, the adjusted HR of RFI for 3-year mortality was 2.03 (95% CI, 1.82–2.27; *P *< 0.001). Figure 1 shows the cumulative survival plot for HE patients with and without RFI. Other factors positively associated with 3-year mortality of HE patients included HCC (HR 2.18; 95% CI, 2.02–2.37; *P* < 0.001), EVB (HR 1.25; 95% CI, 1.13–1.39; *P *< 0.001), age (HR 1.02; 95% CI, 1.00–1.02; *P* < 0.001), male gender (HR 1.09; 95% CI, 1.00–1.17; *P* = 0.042), bacterial infection (HR, 1.32; 95% CI, 1.22–1.84; *P* < 0.001), and ascites (HR, 1.48; 95% CI, 1.38–1.59; *P* < 0.001). Alcohol-related cirrhosis (HR 0.84; 95% CI, 0.77–0.91; *P* < 0.001) was negatively associated, and PUB (HR 0.94; 95% CI, 081–1.10; *P* = 0.439) was not associated with the 3-year mortality of HE patients.

**TABLE 2:**
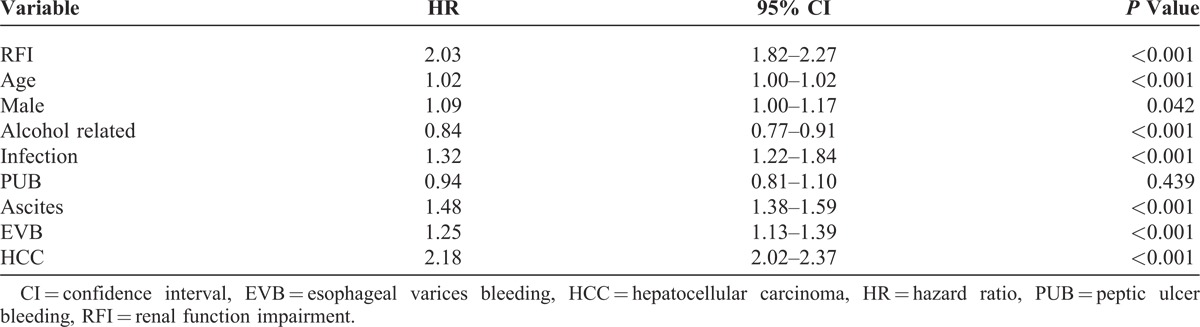
Adjusted Hazard Ratios for 3-Year Mortality in Cirrhotic Patients With Hepatic Encephalopathy

**FIGURE 2:**
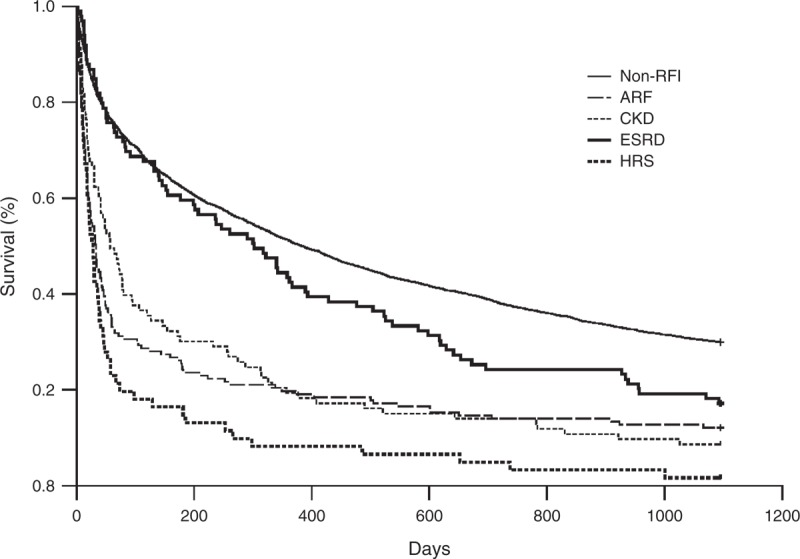
Cumulative survival plot for cirrhotic patients having hepatic encephalopathy with acute renal failure (ARF), hepatorenal syndrome (HRS), end-stage renal disease (ESRD), and chronic kidney disease (CKD), and those without renal function impairment (RFI).

In the RFI group, there were 157 (38.2%) patients with ARF, 61 (14.8%) with HRS, 93 (22.6%) with CKD, and 100 (24.3%) with ESRD. After Cox regression model, the results of HRs of subgroups for the 3-year mortality of HE patients are shown in Table 3. Compared with the non-RFI group, the adjusted HRs of ARF, CKD, HRS, and ESRD was 2.57 (95% CI, 2.17–3.06; *P* < 0.001), 1.93 (95% CI, 1.55–2.40; *P* < 0.001), 3.58 (95% CI, 2.78–4.63; *P* < 0.001), and 1.26 (95% CI, 1.01–1.57; *P* = 0.044), respectively. Figure 2 shows the cumulative survival plot for HE patients with ARF, HRS, ESRD, and CKD. Interestingly, we found that CKD patients had higher HR than ESRD patients.

**TABLE 3:**
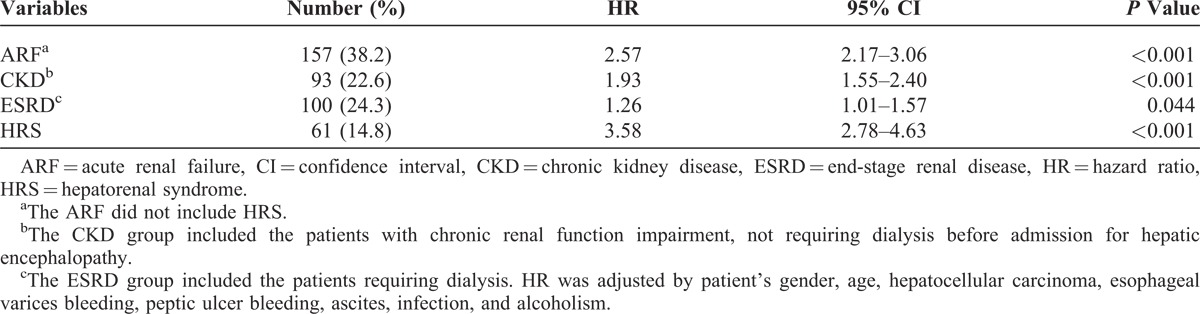
Adjusted Hazard Ratios of Different Types of Renal Function Impairment for 3-Year Mortality of Cirrhotic Patients With Hepatic Encephalopathy Compared With Nonrenal Function Impairment Group

In the ARF group, there were 32 patients receiving hemodialysis in their hospitalizations. Compared with the ARF without hemodialysis, the adjusted HRs of ARF with hemodialysis for the 3-year mortality was 1.278 (95% CI, 0.813–2.008; *P* = 0.287). Hemodialysis for ARF in HE patients did not show a better survival. In HRS group, all patients received standard treatment for HRS, including terlipressin and albumin, and no one received hemodialysis in their hospitalizations. In ESRD group, only 1 patient received peritoneal dialysis and the remaining 99 patients received hemodialysis. In order to identify if ESRD patients with hemodialysis have a better survival than CKD patients, we performed Cox regression model adjusted by age, gender, and other comorbid disorders in HE patients with ESRD and CKD. Compared with the CKD, the HR of ESRD with hemodialysis for the 3-year mortality was 0.664 (95% CI, 0.466–0.945, *P* = 0.023) (Table 4).

**TABLE 4:**
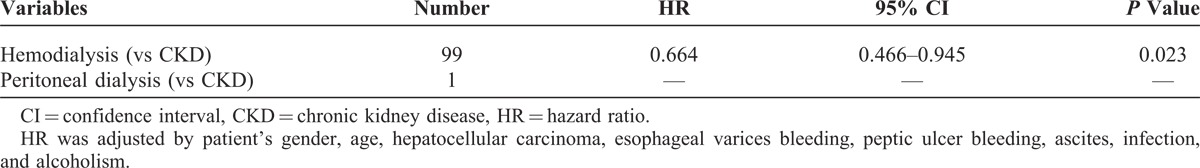
Adjusted Hazard Ratios of Dialysis for 3-Year Mortality of Cirrhotic Patients Having Hepatic Encephalopathy With End-Stage Renal Disease Compared to Those With Chronic Kidney Disease

## DISCUSSION

To our best knowledge, this is the first nationwide population-based study to identify the effect of RFI on the outcomes of cirrhotic patients with HE. Using the nationwide population-based database to enroll a large population of HE patients, our study provides reliable information and reflects the real mortality in recent clinical practice. In previous studies, RFI has been confirmed to be an important predictor of the mortality of cirrhotic patients with spontaneous bacterial peritonitis.^17,23,24^The current study shows that RFI is also an important unfavorable factor for the mortality of cirrhotic patients with HE. The HE patients with RFI had a 2-fold increase in the 3-year mortality, and 90% of them died within 3 years. The other predisposing factors for the mortality of HE patients were age, ascites, HCC, bacterial infections, and EVB. This effect of RFI may be attributed to poor clearance of bloodstream ammonia that increases the susceptibility to brain edema in cirrhotic patients with HE.^11–13^

In this study, we separated HRS from other forms of RFI. HRS is a very specific form of RFI in cirrhotic patients. It is caused by splanchnic arterial vasodilation in cirrhotic patients, and featured by a marked renal vasoconstriction with a consequent reduction in renal plasma flow and glomerular filtration rate, absence of pathologic changes in the kidney tissue, and intact activity of renal tubules.^25,26^ The clinical diagnosis of HRS was based on the criteria established by the International Ascites Club.^27^ We found that the effect of HRS and ARF on the mortality of cirrhotic patients with HE was similar within first 1 month. However, unlike ARF group, the slope of survival curve of HRS did not become gentle after 1 month. This phenomenon reflects that the effect of HRS was more persistent than ARF in cirrhotic patients with HE.

Interestingly, the current study showed that the HE patients with ESRD receiving hemodialysis had better survival than the HE patients with CKD. Theoretically, the CKD patients had better reserve of renal function than the ESRD patients. The formers were speculated to have better survival than the latters. However, the result did not agree this ratiocination. We consider that hemodialysis plays an important role for the better survival in HE patients with ESRD. In HE patients, arterial ammonia concentration is correlated with occurrence of cerebral herniation and increases of intracranial pressure.^28^ The patients treated with dialysis, including peritoneal dialysis, hemodialysis, and hemofiltration, can eliminate water-soluble substances, including ammonia, creatinine, and urea. In previous studies, standard hemodialysis and charcoal hemoperfusion have both been shown to lower serum ammonia levels and lead to clinical improvement in patients with hyperammonemic encephalopathy.^29,30^ This may be reason why HE patients with ESRD receiving hemodialysis had better survivals than those with CKD. However, it needs further prospective study to prove this theory.

Different hospital levels have different therapeutic strategies for cirrhotic patients with HE, which may have some impacts on the mortality of HE patients. Our study is the first nationwide study to identify the effects of RFI on the mortalities of cirrhotic patients with HE. The used database included all HE patients hospitalized in a variety of hospitals in Taiwan. This can prevent selection bias caused by the single-center study. We also reported an overall mortality for cirrhotic patients with HE, including both in and out-of-hospital mortality. This prevented underestimating the mortality, which is possible in studies that include only in-hospital mortality. Nonetheless, there were several limitations in our study. First, although the severity of cirrhosis was commonly based by Child-Pugh score or Mayo Clinic model for end-stage liver disease score, it was not possible to identify the laboratory data such as bilirubin, albumin, prothrombin time, or creatinine by ICD-9 coding numbers in this database. However, the Child-Pugh or Mayo Clinic model for end-stage liver disease score in HE patients was not shown to be associated with outcome in previous studies.^31^ Second, about 31.5% of cirrhotic patients in our study were alcohol related. However, the exact etiology of nonalcoholic liver cirrhosis could not be identified in this population-based study. However, the etiology of nonalcohol-related cirrhosis in Taiwan is mostly known to be related to the hepatitis B virus and has been well established in the previous published reports,^32^ and the etiology of nonalcoholic liver cirrhosis cannot be confirmed as a prognostic factor on survival in cirrhotic patients with HE. Finally, the grade of HE was not possible to be evaluated in this database. However, the grade of HE seems not to be an independent prognostic factor in in-hospital mortality.^7,31^ Udayakumar et al^31^ reported that mild or severe grade of encephalopathy did not predict the mortality in chronic liver disease. In the report by Shawcross et al,^7^ patient survival stratified according to the degree of encephalopathy grade 3 or 4 also did not reveal statistically difference.

Despite these limitations, this nationwide population-based study identified RFI as an important risk factor for the 3-year mortality of cirrhotic patients with HE. RFI increases 2-fold of the mortality in cirrhotic patients with HE. In HE patients with RFI, the HRS patients had the worst outcome. The HE patients with ESRD receiving hemodialysis had better survival than those with CKD.
